# Development of routing algorithms in networks-on-chip based on two-dimensional optimal circulant topologies

**DOI:** 10.1016/j.heliyon.2020.e03183

**Published:** 2020-01-15

**Authors:** Aleksandr Yu. Romanov, Evgeny V. Lezhnev, Aleksandr Yu. Glukhikh, Aleksandr A. Amerikanov

**Affiliations:** National Research University Higher School of Economics, 34 Tallinskaya Ulitsa, Moscow, 123458, Russian Federation

**Keywords:** Electrical engineering, Computer architecture, Computer simulation, High performance computing, Network (Computer science), Algorithms, Network-on-chip, Circulant topology, Two-dimensional optimal circulant topology, Regular topology, Routing algorithm, Multiplicative circulant, Ring circulant

## Abstract

This work is devoted to the study of application of new topologies in the design of networks-on-chip (NoCs). It is proposed to use two-dimensional optimal circulant topologies for NoC design, and it is developed an optimized routing algorithm with the decreased memory usage. The proposed routing algorithm was compared with Table routing, Clockwise routing, and Adaptive routing algorithms, previously developed for ring circulant topologies, and specialized routing algorithm for multiplicative circulants. The results of synthesis of routers implementing proposed routing algorithms are presented. The cost of ALM and register resources for the implementation of communication subsystems in NoCs with circulant topologies is estimated.

## 1 Introduction

Multiprocessor systems-on-chip (MPSoCs) [1] are currently one of the fastest growing areas in computing. An extensive increase in the size of the chip and the number of transistors per unit area allows developing chips with tens and hundreds of processor cores even now. At the same time, the problem of effective organization of a communication subsystem that would ensure quick data exchange between nodes of such NoCs is becoming urgent [2].

Since communication is carried out within the framework of a single chip, laws and principles that exist in telecommunication networks work differently in NoCs; therefore, the development of new solutions and methods for organizing data transfer in such networks is required. In particular, it is the topology that has a significant impact on NoC performance. The most common are classical regular topologies such as mesh [3] and torus [4], which do not always meet modern requirements for NoCs, especially with an increase in the number of nodes [5]. Different researchers suggest other topology options among which we can distinguish hypercube [6, 7, 8], chordal ring [9, 10], spidergon [11], L-networks [12]. Their feature is that they can be reduced to presentation in the form of ring-like graphs called circulant topologies in the general form. Optimal circulants have several significant advantages over mesh and torus topologies – better structural survivability, reliability, and connectivity [13, 14]. Moreover, the mathematical aspects of the theory of circulant graphs are studied quite well; such families of graphs as recursive circulants [15], multiplicative circulants [16], ring circulants [14] having quite interesting properties are known. The previously mentioned topologies (hypercube, chordal ring, etc.), in theory, are also close to circulant topologies and often are a special case of circulant subfamily, but of a holistic consideration of circulant networks as topologies for developing NoCs has not been fully made before. It is necessary to study circulant topologies and routing algorithms in them taking into account the chip resources necessary to implement such NoCs.

## 2 Background

### 2.1 Two-dimensional circulants

**Figure 1:**
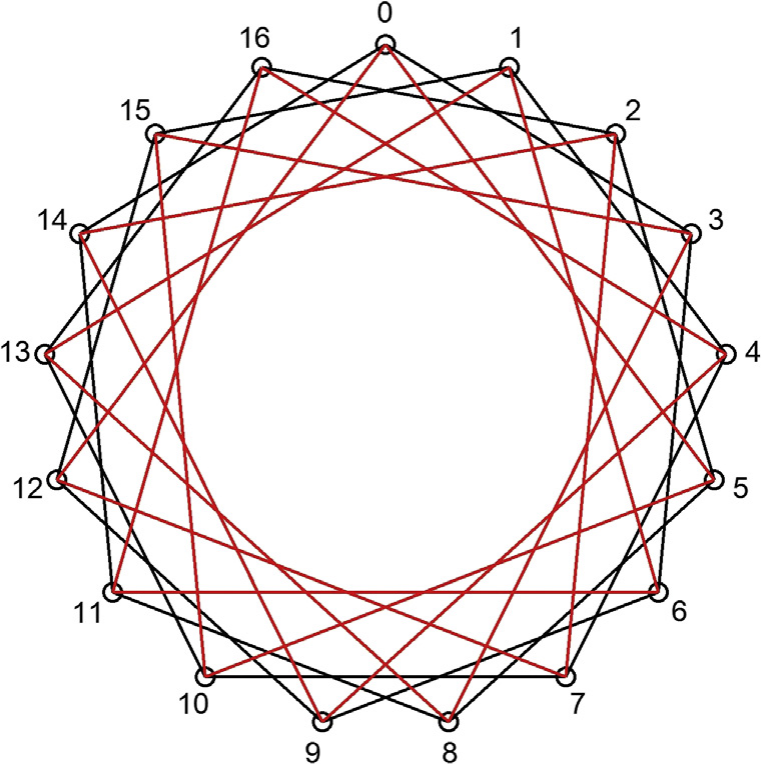
Circulant graph $C\left(17;2,5\right)$.

A non-oriented graph with set of vertices $V=\left\{1,...,N-1\right\}$ and set of edges $E=\left\{\left(I,j\right):\left|I–j\right|\equiv s_{m}\;\text{mod}\;N,m=1,\ldots ,k\right\}$ is called a circulant graph [13] ($S=s_{1},s_{2},\ldots ,s_{k}$ – generators, N – number of graph vertices) (Figure 1). The signature $C\left(N;s_{1},s_{2},...,s_{k}\right)$ is a representation of a circulant as a ring-like regular structure, where every vertex is connected by chords $s_{1},\ldots ,s_{k}$ with successive and previous vertices.

The most common are two-dimensional circulants of type $C\left(N;s_{1},s_{2}\right)$ which have the same degree of vertices as mesh and torus topologies. This allows the use of routers with 4 inputs/outputs previously developed by other researches (for example, Netmaker library [17]).

The main characteristic of any graph is its diameter: $d\left(C\right)=\underset{i,j\in V}{\max}\;\text{len}\left(i,j\right)$, where len(i,j) – length of the shortest path from vertex i to vertex j of graph C. Synthesis of optimal graphs with minimal diameter is a fundamental problem of graph theory. For some types of circulants ($C\left(N;D,D+1\right),$ for example), formulas are known for the optimal circulants calculating [18, 19]; for others – the software to find them is developed [20]. Some circulant graphs subclasses like ring circulants $C\left(N;1,s_{2}\right)$ [14], where the first generator equals 1, and multiplicative circulants $MC\left(s,k\right)=C\left(s^{k};1,s,s^{2},\ldots ,s^{k-1}\right)$ [16, 21] are not always extremely optimal, but they have a number of useful properties for using them as topologies for NoCs.

Work [14] shows that circulants (at equal costs of connective resources) have less diameter and average distance in comparison with torus and mesh. There are optimal circulant graphs for any number of vertices with no reduction in their effectiveness. Thus, circulant graphs are the promising topological basis for NoC development to improve NoC characteristics (in comparison with mesh and torus topologies) which keep, simultaneously, regularity of networks and dimension of routers.

## 3 Study area

### 3.1 Routing in two-dimensional circulants

Due to the fact that circulant topologies have a regular structure, routing in them is not as difficult as, for example, in irregular networks [22]. Nevertheless, the development of optimal algorithms that would minimize the time of delivery of packages to the destination nodes is required. With reference to NoCs, communication between nodes can be broadcasting, gossiping and point-to-point.

#### 3.1.1 Broadcasting and gossiping in two-dimensional circulants

The first two types are mainly relevant for homogeneous parallel computing systems [23], where broadcasts from one control node to all the others (broadcasting) or from all the nodes to all the nodes (gossiping) are common; for example, to configure a network and define its structure which may change dynamically. The peculiarity of such exchanges is that the broadcast packets are simply transmitted along the chain from one node to all the neighboring ones, and so on until they reach all the nodes of the network. It is enough to ensure that one packet, once in the same node several times, is not perceived as a new one every time, as well as the lifetime of the packets in the network, so that they are not transmitted infinitely. Such packet exchange processes are mainly described using communication models and are well studied for circulant networks. For example, in [19] a shouting model is presented, according to which each vertex exchanges with its neighbors only once. To eliminate packet duplication at each transit vertex, routing is performed on the basis of the spanning tree with the shortest paths centered at this vertex. Thus, the delivery time of packages is equal to the diameter of the graph.

For NoCs, the shortest path tree is rather redundant in each router, so one can use algebraic approaches to save resources [24]; in work [25] it was shown that for a simplified one-port model (whispering model), when one node at a time can exchange with only one neighboring node, the broadcasting time does not exceed the diameter value +2.

The gossiping task is more complicated, but in general it is also solved by distributing approaches for broadcasting to all the network nodes. For the particular case of two-dimensional circulants called Gaussian networks, the work [24] proposed a fast algorithm for all-to-all broadcast which was applied to NoCs using additional buffers in routers [26] and was improved in [27].

For another particular case of two-dimensional circulants [28], the problem was solved in [29] by constructing optimal trees for translational exchanges, and in [30], the *i*-port model (i≤4) for the graphs of type $C\left(N;D,D+1\right)$ was presented.

#### 3.1.2 Point-to-point communication in two-dimensional circulants

The most important communication problem for any network is the organization of point-to-point communication between two vertices. The most universal and well-known algorithm for finding the shortest path between two vertices is the Dijkstra algorithm [31]. It has complexity $\text{O}\left(n^{2}\right)$ and is redundant for circulant graphs. Therefore, for various subclasses of two-dimensional circulants, there is a number of developments in routing algorithms. Thus, in [32] an algorithm of complexity $\text{O}\left(D\right),\text{where }D-$diameter, was proposed for ring circulants. The issues of pair exchanges in multiplicative circulants are considered in [9, 16]. For optimal graphs of type $C\left(N;D,D+1\right)$, as well as $C\left(N;1,2D+1\right)$, in work [33], a method for constructing spanning trees was proposed.

A universal algorithm for any two-dimensional circulants, having O(D) complexity and ensuring that the packet reaches the destination node in l=O(D) steps, is given in [24]. Another work [34] presented an algorithm for finding a shortest path between two vertices of any weighted undirected and directed two-dimensional circulant graph. It requires O(logN) arithmetic steps and its total bit complexity is $\text{O}\left({\text{log}}^{3}N\right)$. But the work [19], where it is given a description of the algorithm for finding paths in the graphs $C\left(N;D,D+1\right)$ which have complexity O(1) and guarantee finding of the optimal path, is of much interest.

#### 3.1.3 Problems of routing in networks-on-chip based on circulant topologies

Despite the fact that there are already quite a few different algorithms for routing in circulant networks, there are quite a few limitations determined by the fact that they are to be applied to NoCs. The problem of NoCs is that the communication subsystem in them often occupies tens of percent of the chip area [35], and power consumption may reach 36 % [36].

It may be of interest (in the context of the above) to use the interval routing method in circulant networks described in works [37, 38]. This approach allows storing routing tables in a compact form (O(logN) bits each) in every vertex of the graphs of type $\left(N;1,s_{2}\right)\text{and }C\left(N;1,2D+1\right)$ . Moreover, like any algorithm, based on the routing tables, complexity of the routing algorithm is equivalent to O(1). However, this approach still does not solve the problem, since there is a need to develop routing algorithms implemented as RTL state machine in routers for NoCs. RAM blocks, DSP blocks and ALMs utilization should be minimized. In this case, there is no need to calculate the entire path of the packet, but calculate the port number to which the packet should be sent so that it can reach the destination node. This makes it possible to significantly simplify the structure of the NoC router.

## 4 Design

### 4.1 Description of the routing algorithm for C(N;D,D+1) circulants

It was first proven in [39] and then confirmed once again in [18] that circulants with a parametric description of $C\left(N;D,D+1\right)$ are optimal in diameter and average distance; finding parameter D is performed according to formula:

(1) $$C\left(N;D,D+1\right),\text{ where }D=\sqrt{N/2}-1,N>2$$

Unlike circulants $C\left(N;1,s_{2}\right)$, in circulants $C\left(N;D,D+1\right)$, in order to pass to the neighboring node, it is necessary to take 2 hops (first, along generator $s_{2}$, then back along $s_{1}$). Let's call them “single” hops (Figure 1). “Single” hops are made as follows: along $s_{2}$, it is necessary to go towards the top; along $s_{1}$ – to the opposite side. If hops to only neighboring nodes are made, and the whole cycle is gone through in a similar way, exactly 2N hops will be required, that is why it is necessary to consider situations so that it could be avoided.

Any calculations of the distance between the final and initial node in this type of circulants are reduced to the following formula:

(2) $$hops=\left|x\right|+\left|y\right|,length=x•s_{1}+y•s_{2}$$

where x,y∈Z, hops – number of hops required to pass from the initial node to the final node along generators $s_{1}$ and $s_{2}$;

length – internodal distance.

For convenience of description of the algorithm, we introduce the notion of clockwise motion. At the first step of the algorithm, it is calculated in which direction the packet is required to be sent and how many hops are required. To do this, subtract index of the end node from index of the initial node. If the distance value turns out to be negative, it is necessary to choose a counterclockwise motion and take absolute value of resulting distance. If this number is greater than half of the network nodes, then, according to the algorithm, it is necessary to turn in the opposite direction and calculate the distance taking this direction into account.

After calculating the length and direction, it is necessary to determine which of the generators is required to take a hop. Trivial choice is in the case when the distance is divided by one of the generators without a residue (3). From this, it follows that it is necessary to choose the next vertex, based on the sum or difference of index of the current node and value of the selected generator. The exception is the situation when the length exceeds the product of values of the two generators. In this case, the path can be considered to be optimal in distance:

(3) $$hops=length/{\text{s}}_{1}\;\text{or}\;hops=length/s_{2}$$

If the current distance does not meet above mentioned conditions, it is necessary to consider cases when it is possible to get as close as possible to the end node by choosing hops for one or another generators. This strategy can give a better result than the use of “single” hops. The next assumption is that the number of hops required to cover the distance $n•s_{1}$ and $n•s_{2}$ will be equal to n, since nothing prevents to choose one or another vertex. Suppose n is an integer division of the distance by $s_{1}$; in this case, the number of hops will be equal to n, if the distance does not exceed the value of $n•s_{2}$.

(4) $$n•s_{1}\leq length\leq n•s_{2}$$

where $n=length/s_{1}$ – integer.

This condition is well suited for finding a path if the distance does not exceed $s_{1}•s_{2},$ and the number of hops required can cover the distance. It should be taken into account the previously introduced restriction that the distance should not exceed half of the number of nodes.

Consider the statement that there is a possibility to pass the distance in n+1 hops (where 1 is not a “single” hop, but an additional hop along generator $s_{1}$; n is the integer from dividing distance by $s_{2}$). However, the following condition must be observed:

(5) $$length/s_{2}+length\text{ \% }s_{2}\geq s_{1}$$

Under this condition, it will be necessary to choose $s_{1}$ until $length\;\%\;s_{2}>0$. After this, the algorithm will move along $s_{2}$.

To implement the algorithm, it is necessary to choose one of the two above conditions.

Consider a case that does not fit all the above conditions. It turns out that if we go along one or another generator, the remaining distance (in this case, $length<s_{1}$) must be reached by “single” hops. In some cases, the optimal strategy is to take an additional hop in order to avoid unnecessary “single” hops. Taking generator $s_{1}$ as the basic one, the formula for finding the need to take an additional hop will be as follows:

(6) $$length<\left|length–s_{1}\right|$$

If the condition is true, then an additional hop is taken along generator $s_{1}$.

We introduce another condition that the distance is not greater than $s_{2}$; it needs to be checked in order to avoid extra “single” hops, since there may be a situation when the calculated distance will be longer in a particular range. For example, in this case:

(7) $$n\cdot s_{2}<X<s_{1}/2<Y<\left(n+1\right)\cdot s_{1}$$

where X – non-optimal value of the distance, if the algorithm moves along $s_{1}$ first and then does “single” hops; Y – the same with $s_{2}$, respectively.

Thus, to determine which generator is worth choosing, one must perform the following steps for each generator:

1. Calculate the division of the current distance by generator. The residue will be considered to be the remaining distance (last_length); the integer – the number of hops taken.
2. Check the condition ($last\_length<\left|last\_length–s_{1}\right|$); if it is satisfied – 1 hop is taken, if not – 0 hops.
3. When a hop is taken in the previous step, the number of remaining hops is calculated as $\left|last\_length–s_{1}\right|\cdot 2$; otherwise –2·last_length.
4. Sum hops of the previous three stages of algorithm.

The obtained sums for two generators must be compared. If it turns out that $s_{1}$ sum is less than $s_{2}$ sum the hop along $s_{1}$ is taken; otherwise – along $s_{2}$.

For the operation of the algorithm, the head fleet should contain the number of the destination node. The load on the packet for N nodes in the network is calculated by the following formula:

(8) $$P={\lceil \text{log}}_{2}N\rceil$$

In the router, it is necessary to store its number in the network, the total number of routers in the network, as well as length of small generator (D). Thus, the size of the stored data in the router can be estimated using the following formula:

(9) $$M_{base}=2•\left\lceil \log_{2}N\right\rceil +\lceil \log_{2}\frac{N}{2}\rceil +2$$

where $\left\lceil \log_{2}N\right\rceil$ – memory (in bits) to store the sequence number of the router and the total number of routers in the network;

$\left\lceil \log_{2}\frac{N}{2}\right\rceil$ – memory (in bits) to store generator D;
2 – memory (in bits) for storing generators selection flags for transmitting the packet to the next node.

We store only generator D, since the second generator, based on the parametric description of this type of circulants, can be easily calculated as D+1, and storing it does not make sense. Also, to store the value of generator D, exactly $\left\lceil \log_{2}\frac{N}{2}\right\rceil$ bits is required, since generator D is guaranteed to be less than half of number of nodes which follows from the symmetry of circulant graphs.

## 5 Calculation

For the proposed algorithm to work, it is necessary to store intermediate data for calculations, so total size of the stored data by router can be estimated using the formula:

(10) $$M_{alg}=M_{base}+2•\left\lceil \log_{2}N\right\rceil +3•\lceil \log_{2}\frac{N}{2}\rceil +2$$

To get the total amount of data stored in the network it is necessary to multiply $M_{alg}$ by the number of routers in the network.

Based on the above formulas, values of the data, stored in the network (in bits), were theoretically obtained (Table 1).

**Table 1:** Memory resources (in bits) taken by the algorithm for circulants of type $C\left(N;D,D+1\right)$.

Circulant	Number of nodes	Diameter	1 router, bits	Whole network, bits
C(9;2,3)	9	2	28	252
C(16;2,3)	16	3	28	448
C(25;3,4)	25	3	40	1000
C(36;4,5)	36	4	48	1728
C(49;4,5)	49	5	48	2352
C(64;5,6)	64	6	48	3072
C(81;6,7)	81	6	56	4536
C(100;7,8)	100	7	56	5600

**Figure 2:**
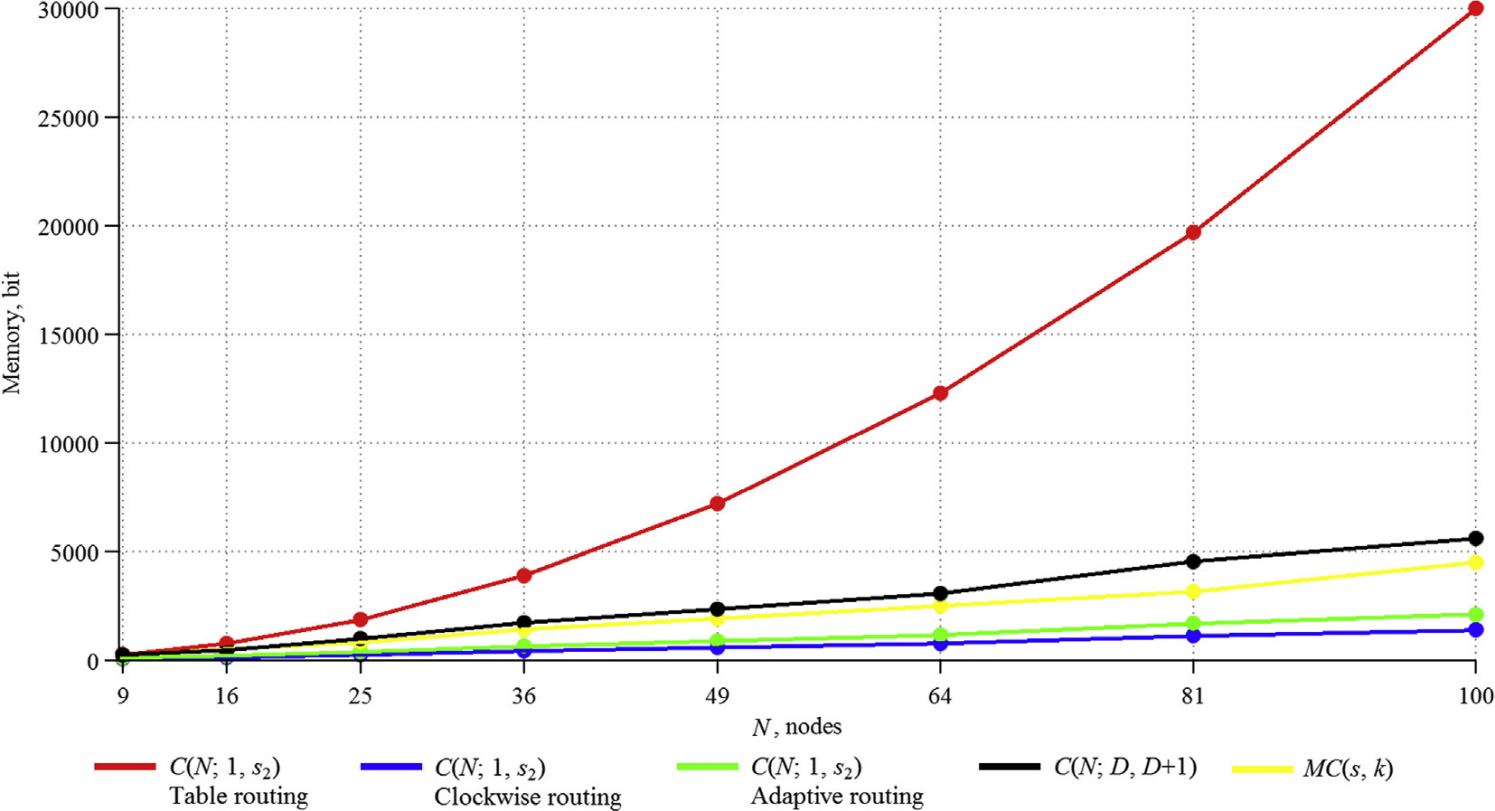
Memory resources taken by the algorithms for considered two-dimensional circulants.

**Figure 3:**
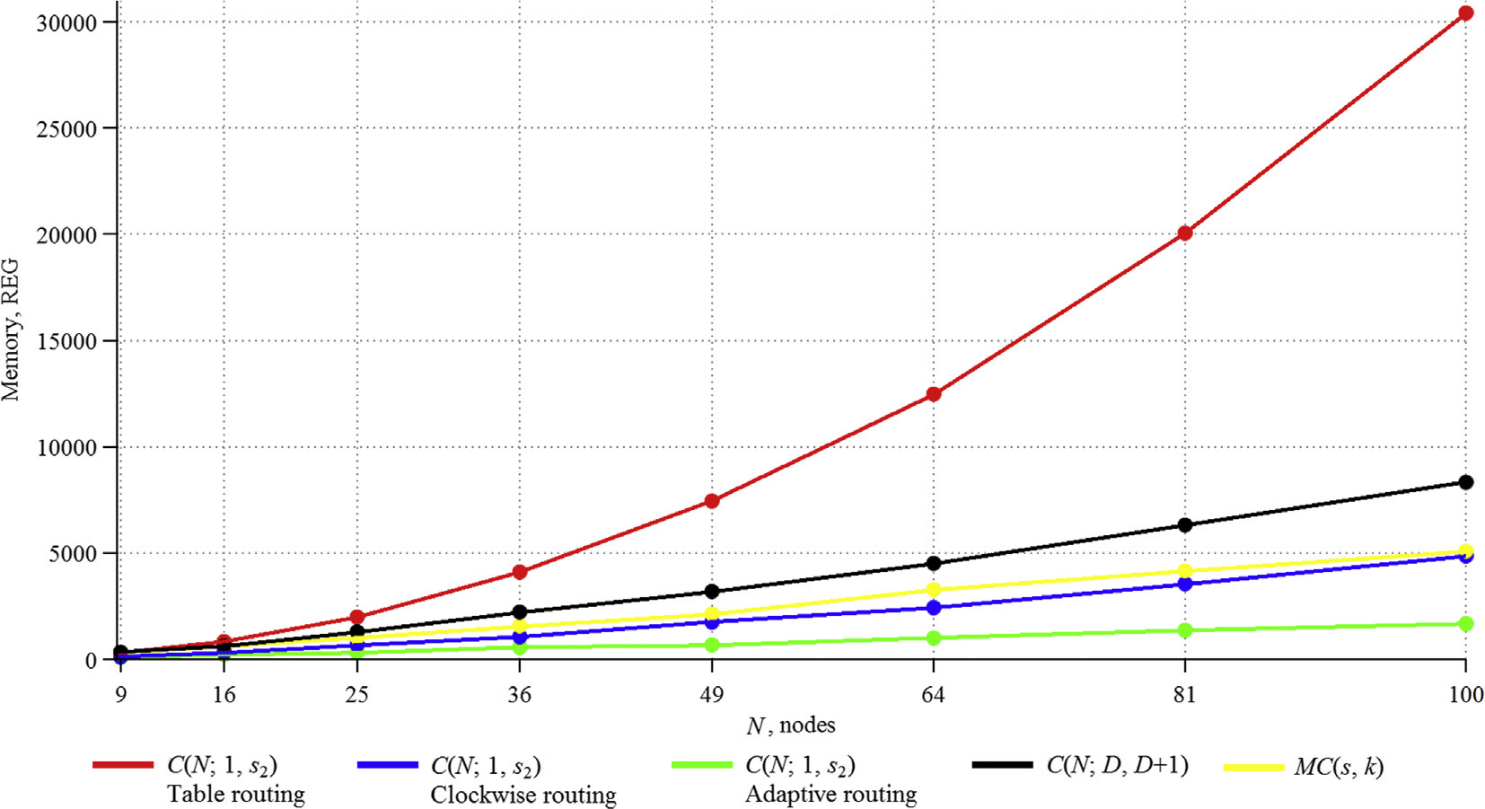
Dependence of memory, consumed by algorithms, on network size.

**Figure 4:**
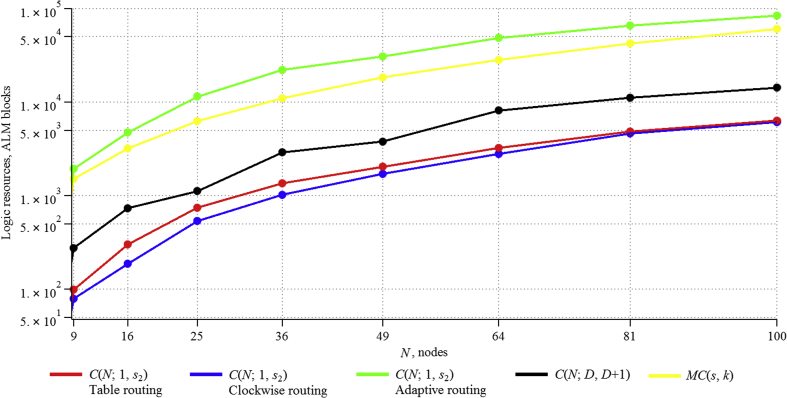
Dependence of ALM blocks, consumed by algorithms, on network size.

Thus, it can be concluded that the required number of bits for storing routing information in $C\left(N;D,D+1\right)$ circulants is fully consistent with the current characteristics of modern chips, for example, Cyclone V5CGXFC9A6U19I7 [40].

## 6 Experimental results

### 6.1 Comparison with routing algorithms for two-dimensional circulants of other types

#### 6.1.1 Algorithms for circulants of type $C\left(N;1,s_{2}\right)$

For circulants of type $C\left(N;1,s_{2}\right)$ [13], several routing algorithms were proposed [14]. The first algorithm (Table routing algorithm) implements routing using special tables that store pre-calculated information about the next hop to deliver the packet to the desired network node. The table is stored in a distributed manner – only the part responsible for routing from a given node is stored in each router. In fact, implementation of such an algorithm is the simple finite-state machine. The input of the algorithm receives the number of the destination node stored in the address field of the head flit package. From the routing table, by the destination node number, a value, that indicates which port of the router the incoming packet should be sent to, is selected. The load on the packet for circulants $C\left(N;1,s_{2}\right)$ is similar to the developed above algorithm (8). Total amount of memory, needed to implement the algorithm, can be estimated by the formula:

(11) $$M_{table}=N^{2}•\lceil \log_{2}p\rceil$$

where $\left\lceil \log_{2}p\right\rceil$ – memory (in bits) to store the port number of the router;

p – number of router ports (p=4 for two-dimensional circulants).

The Table routing algorithm is universal and suitable for any topology, but at the same time, amount of memory, consumed for its implementation, grows in quadratic dependence on the number of nodes [14].

The second algorithm (Clockwise routing algorithm) implements an iterative calculation of route of the source node to the destination node. Each router calculates only 1 next routing hop. The load on the package also coincides with the developed algorithm. For Clockwise routing algorithm to work, each router needs to store several values characterizing the graph used. The size of stored data can be estimated by the formula:

(12) $$M_{clockwise}=N\text{*}\left(\left\lceil \log_{2}N\right\rceil +\lceil \log_{2}\frac{N}{2}\rceil \right)$$

where $\left\lceil \log_{2}N\right\rceil$ – memory (in bits) to store the number of routers in the network;

$\left\lceil \log_{2}\frac{N}{2}\right\rceil$ – memory (in bits) to store generator $s_{2}$.

The algorithm works as follows: first, the next hop direction is determined; it coincides with, or is opposite to the direction of clockwise movement. If the difference between the source and destination nodes of the packet is greater than N/2, then direction of transmission of the packet is selected clockwise; otherwise – counterclockwise. After selecting the direction of movement, the packet is transmitted along the larger generator towards the receiver node until the difference between the nodes is smaller than the size of the larger generator. After that, reaching the destination node along single generators occurs. This algorithm is not optimal because of the number of hops relative to diameter of the network. The termination criterion for a packet is a zero value in address field of the head flit.

The third algorithm (Adaptive routing algorithm) is an improvement of the previous algorithm, while it finds the optimal route relative to the network diameter. The size of the data, stored by the algorithm, can be estimated by the formula:

(13) $$M_{adaptive}=N\text{*}\left(2\text{*}\left\lceil \log_{2}N\right\rceil +\lceil \log_{2}\frac{N}{2}\rceil \right)$$

where $\left\lceil \log_{2}N\right\rceil$ – memory (in bits) to store the router number and number of routers in the network;

$\left\lceil \log_{2}\frac{N}{2}\right\rceil$ – memory (in bits) to store generator $s_{2}$.

In order for the algorithm to calculate all the data in a non-negative form, first, the sequence of transferring node numbers to the main block of the algorithm is selected. This possibility arises due to the fact that the graph is undirected and vertex-transitive as well [41]. Then it is calculated how many hops needed to be done along the larger generator to the destination node. Two options are considered: if a transition was made via the destination node or not. Then, the number of “single hops” to be taken to get to the destination node is added to the obtained values. Such calculations are done both when moving clockwise or counterclockwise. Then it is checked which variant has fewer hops.

Also, in this algorithm, the notion of “cycle” is introduced. A cycle is understood as such a movement along the greater generator which leads to the passage via the node of the reference point of the cycle. Thus, there are two additional variants of possible movement along the larger generator. As a result, the packet is transmitted in the direction for which calculations reached the minimum required number of hops to the destination node.

#### 6.1.2 Routing algorithms for circulants of typeMC(s,k)

For circulants of type $MC\left(s,k\right)$ [16], a specialized algorithm [21], which takes into account the geometric features of multiplicative circulants – lengths of generators being degrees from 0 to k−1 with the base s, was proposed. The number of destination node is also transmitted in the address field. The size of stored data in the network for can be calculated by the formula:

(14) $$M_{MC\left(s,k\right)}=N\cdot \left(\left\lfloor {\text{log}}_{2}N\right\rfloor +\left\lceil {\text{log}}_{2}N\right\rceil +k\cdot \left(\left\lfloor {\text{log}}_{2}s^{k-1}\right\rfloor +1\right)+3\cdot \left\lceil {\text{log}}_{2}k\right\rceil +2\right)$$

where $\left\lfloor {\text{log}}_{2}N\right\rfloor +1$ – memory to store the number of routers in the network;

$\left\lceil {\text{log}}_{2}N\right\rceil$ – memory to store the router number;
$k•\left(\left\lfloor {\text{log}}_{2}s^{k-1}\right\rfloor +1\right)$ – memory to store the array of generators;
$\left\lceil {\text{log}}_{2}k\right\rceil$ – memory to store the indices of generators and primary port of the router;
1 – memory to store the flag of primary port.

Algorithm can be described as follows [21]: first, the current node number is recalculated, and the primary direction of packet transmission is selected (clockwise or counterclockwise). Then, the recalculated node number is compared with generators (starting from the larger generator), and the index of the first generator, which is greater than the recalculated node number, is recorded. Next, the comparison of results of displacement along the selected generator with the second remaining one is made. The generator, that is closest to the destination node, is chosen.

Table 2 summarizes the results of theoretical calculations of consumed memory resources for routing algorithms in ring and multiplicative graphs.

**Table 2:** Memory resources (in bits) taken by the algorithm for circulants of type $C\left(N;1,s_{2}\right)$ and $MC\left(s,k\right)$.

Circulant	Number of nodes	Table routing algorithm	Clockwise routing algorithm	Adaptive routing algorithm	Multiplicative routing algorithm
C(9;1,3)	9	243	72	108	243
C(16;1,6)	16	768	128	192	528
C(25;1,5)	25	1875	250	375	825
C(36;1,8)	36	3888	432	648	1404
C(49;1,9)	49	7203	588	882	1911
C(64;1,14)	64	12288	768	1152	2496
C(81;1,24)	81	19683	1134	1701	3159
C(100;1,18)	100	30000	1400	2100	4500

Thus, it can be noted that Table routing uses many times more memory than all the algorithms considered earlier. The algorithm, developed for C(N;D,D+1) circulants, uses a comparable amount of resources with MC(s,k), and it is several times larger than the Clockwise routing and Adaptive routing algorithms. The use of the Adaptive routing algorithm is better than Clockwise routing, because it has greater efficiency [14].

Theoretical calculations of memory usage for the implementation of all considered algorithms are presented in the cumulative diagram (Figure 2, Tables 1 and 2). Full supplementary data are provided in Appendix 1 (Fig. A1–A5, Tables A1–A5).

### 6.2 Testing of routing algorithms for circulants of various types

The approbation of the proposed algorithm for circulants of type $C\left(N;D,D+1\right)$, was carried out on FPGA Cyclone V5CGXFC9A6U19I7 [40] of Intel FPGA (Altera). Routers were implemented in Verilog. For testing, we selected optimal circulants of type $C\left(N;D,D+1\right)$ with the number of nodes defined by the formula $N=m^{2}$, where N – number of nodes of the network, m – natural number. This restriction on selection of graphs is justified by the possibility of comparing the resources occupied by algorithms for different topologies of circulants, as well as comparing with optimal mesh and torus topologies having the same number of nodes.

On the graph of theoretical calculation of the used memory for storing network data and the graph of simulation results, one can notice a discrepancy of 20–30 % in increasing the used memory. This discrepancy between the calculations occurs due to the use of additional logic for testing the network operation on FPGA for whose simulation logical and memory resources are also needed.

For the proposed algorithm, a comparison on the resources, occupied by algorithms for circulant topologies and other parametric descriptions, was made. Since circulants, considered in the paper, are two-dimensional circulants $C\left(N;D,D+1\right)$, $C\left(N;1,s_{2}\right)$ and $MC\left(s,k\right)$ were also chosen for comparison. It should be noted that while the circulants $C\left(N;D,D+1\right)$ were selected for simulation not only by the number of nodes, but also with a minimum diameter and average distance between nodes (optimal ones), the topology with descriptions $C\left(N;1,s_{2}\right)$ and $MC\left(s,k\right)$ was not always optimal.

This study did not analyze the bandwidth capability and the average packet delivery delay in the network achieved using these algorithms, since it required that modeling using special models [42, 43, 44] be carried out which is a rather time-consuming procedure and is subject for separate consideration. Indirectly, one can evaluate these parameters by how much the optimal path for the packets provides the algorithm. In this case, all algorithms except Clockwise are optimal [18, 21, 39] therefore, it can be argued that their timing parameters will be close, although this requires further study.

The results can be presented as summary (Figures 3 and 4; full data are provided in Appendix 2: Figures B1–B10, Tables B1–B5):

The obtained experimental data confirm the theoretical calculations, which allows using them for networks with a large number of nodes. The proposed algorithm for circulants $C\left(N;D,D+1\right)$ is worse than Adaptive routing and the algorithm for multiplicative circulants on consumed registers, but it is better at ALM blocks. Moreover, the Adaptive routing algorithm needs to add additional logic to eliminate loops with an increase in the number of nodes [14], which greatly affects the resources consumed, and on a scale of thousands of nodes, the difference from the proposed algorithm for consumed registers will be less, and this resource itself is much less important than ALM blocks. Also $C\left(N;D,D+1\right)$ circulants themselves, for some orders, have better characteristics [14], and they are more universal, since their description is given by a formula and does not require a special search procedure [20], while multiplicative circulants exist only for a certain number of nodes [21].

## 7 Conclusion

Based on results of the comparison of algorithms, it can be noted that the algorithm for circulants of type $C\left(N;D,D+1\right)$ with almost the same use of logical resources of the chip uses considerably less registers for storing values than the adaptive algorithm for circulants of type $C\left(N;1,s_{2}\right)$. The algorithm for multiplicative circulants with parametric descriptions $MC\left(s,k\right)$ uses several times fewer resources than the algorithm for circulants of type $C\left(N;D,D+1\right)$, but it has restrictions on the number of network nodes for which it can be used, and also the multiplicative circulants themselves are not always optimal.

If it is necessary to minimize the consumption of logical resources, Table routing algorithm shall preferably be used. But this choice leads to a sharp increase in memory usage for data storage. The use of multiplicative circulants can be a good solution, since it leads to average costs of both logical and memory resources; it also overcomes the shortcomings described earlier.

Thus, in case of consideration of two-dimensional circulants in general, the use of circulants $C\left(N;D,D+1\right)$ is the most universal solution, and the developed routing algorithm expands the possibilities of using such topologies for building NoCs with more than hundreds of nodes.

## Declarations

### Author contribution statement

Aleksandr Yu. Romanov: Conceived and designed the experiments; Performed the experiments; Analyzed and interpreted the data; Wrote the paper.

Evgeny V. Lezhnev: Performed the experiments; Analyzed and interpreted the data; Contributed reagents, materials, analysis tools or data.

Aleksandr Yu. Glukhikh: Performed the experiments; Wrote the paper.

Aleksandr A. Amerikanov: Contributed reagents, materials, analysis tools or data.

### Funding statement

This article is an output of a research project implemented as part of the Basic Research Program at the National Research University Higher School of Economics (HSE University).

### Competing interest statement

The authors declare no conflict of interest.

### Additional information

Data associated with this study has been deposited at GitHub under https://github.com/RomeoMe5/routingAlgorithms_DoubleLoopCirculant, and https://github.com/RomeoMe5/circulantGraphs.

## References

[1] Paul J., Stechele W., Oechslein B., Erhardt C., Schedel J., Lohmann D., Schröder-Preikschat W., Kröhnert M., Asfour T., Sousa É., Lari V., Hannig F., Teich J., Grudnitsky A., Bauer L., Henkel J. (2015). Resource-awareness on heterogeneous MPSoCs for image processing. J. Syst. Archit..

[2] Abdelfattah M.S., Bitar A., Betz V. (2017). Design and applications for embedded networks-on-chip on FPGAs. IEEE Trans. Comput..

[3] Deb D., Jose J., Das S., Kapoor H.K. (2019). Cost effective routing techniques in 2D mesh NoC using on-chip transmission lines. J. Parallel Distrib. Comput..

[4] Ansari A.Q., Ansari M.R., Khan M.A. (2016). Modified quadrant-based routing algorithm for 3D Torus Network-on-Chip architecture. Perspect. Sci..

[5] Dally W.J., Towles B.P. (2003). Principles and Practices of Interconnection Networks.

[6] Marvasti M.B., Szymanski T.H. (2012). The performance of hypermesh NoCs in FPGAs. IEEE Int. Conf. Comput. Des. VLSI Comput. Process..

[7] Saldana M., Shannon L., Bian S., Yue J.S., Craig J., Chow P. (2007). Routability of network topologies in FPGAs, IEEE trans. Very large scale integr. Off. Syst..

[8] Kurihara T., Li Y. (2016). A cost and performance analytical model for large-scale on-chip interconnection networks. Fourth Int. Symp. Comput. Netw., IEEE, 2016.

[9] Parhami B. (2006). A class of odd-radix chordal ring networks. CSI J. Comput. Sci. Eng..

[10] Ajwani D., Hackett A., Ali S., Morrison J.P., Kirkland S. (2016). Co-optimizing application partitioning and network topology for a reconfigurable interconnect. J. Parallel Distrib. Comput..

[11] Bishnoi R., Kumar P., Laxmi V., Gaur M.S., Sikka A. (2014). Distributed adaptive routing for spidergon NoC. 18th Int. Symp. VLSI Des. Test, VDAT 2014.

[12] Camarero C., Martinez C., Beivide R., L-Networks (2013). A topological model for regular 2D interconnection networks. IEEE Trans. Comput..

[13] Monakhova E.A. (2012). A survey on undirected circulant graphs. Discret. Math. Algorithms Appl..

[14] Romanov A.Y. (2019). Development of routing algorithms in networks-on-chip based on ring circulant topologies. Heliyon.

[15] Park J.-H., Chwa K.-Y. (1994). Recursive circulant: a new topology for multicomputer networks. Int. Symp. Parallel Archit. Algorithms Networks (ISPAN 1994).

[16] Stojmenović I. (1997). Multiplicative circulant networks topological properties and communication algorithms. Discrete Appl. Math..

[17] Mullins R., West A., Moore S. (2004). Low-latency virtual-channel routers for on-chip networks. Comput. Architect. News.

[18] Beivide R., Herrada E., Balcazar J.L., Arruabarrena A. (1991). Optimal distance networks of low degree for parallel computers. IEEE Trans. Comput..

[19] Monakhova E.A. (1982). Machine-to-machine interaction algorithms and reconfiguration of connection graphs in computer systems with a programmable structure. Comput. Syst. Quest. Theory Constr. Comput. Syst..

[20] Romanov A.Y., Romanova I.I., Glukhikh A.Y. (2018). Development of a universal adaptive fast algorithm for the synthesis of circulant topologies for networks-on-chip implementations. 2018 IEEE 38th Int. Conf. Electron. Nanotechnol., IEEE.

[21] Shchegoleva M.A., Romanov A.Y., Lezhnev E.V., Amerikanov A.A. (2019). Routing in networks on chip with multiplicative circulant topology. J. Phys. Conf. Ser..

[22] Romanov A.Y., Romanova I.I. (2015). Use of irregular topologies for the synthesis of networks-on-chip. 2015 IEEE 35th Int. Conf. Electron. Nanotechnol., IEEE.

[23] Hedetniemi S.M., Hedetniemi S.T., Liestman A.L. (1988). A survey of gossiping and broadcasting in communication networks. Networks.

[24] Martínez C., Beivide R., Stafford E., Moretó M., Gabidulin E.M. (2008). Modeling toroidal networks with the Gaussian integers. IEEE Trans. Comput..

[25] Liestman A.L.L., Opatrny J., Zaragoza M. (1998). Network properties of double and triple fixed step graphs. Int. J. Found. Comput. Sci..

[26] Zhang Z., Guo Z., Yang Y. (2013). Efficient all-to-all broadcast in Gaussian on-chip networks. IEEE Trans. Comput..

[27] Touzene A. (2015). On all-to-all broadcast in dense Gaussian network on-chip. IEEE Trans. Parallel Distrib. Syst..

[28] Boesch F.T., Wang Jhing-Fa, Wang J.F. (1985). Reliable circulant networks with minimum transmission delay. IEEE Trans. Circuits Syst..

[29] Comellas F., Mitjana M., Peters J.G. (2002). Broadcasting in small-world communication networks. SIROCCO 9, Proc. 9th Int. Colloq. Struct. Inf. Commun. Complex., Andros, Greece.

[30] Obradović N., Peters J., Ružić G. (2005). Reliable broadcasting in double loop networks. Networks.

[31] Deng Y., Chen Y., Zhang Y., Mahadevan S. (2012). Fuzzy Dijkstra algorithm for shortest path problem under uncertain environment. Appl. Soft Comput. J..

[32] Robič B. (1996). Optimal Routing in 2-jump Circulant Networks. https://www.cl.cam.ac.uk/techreports/UCAM-CL-TR-397.html.

[33] Fabrega J., Zaragozà M. (1997). Fault-tolerant routings in double fixed-step networks, Discret. Appl. Math..

[34] Gómez D., Gutierrez J., Ibeas Á., Martínez C., Beivide R. (2005). On finding a shortest path in circulant graphs with two jumps. Int. Comput. Comb. Conf..

[35] Balfour J., Dally W.J. (2014). Design tradeoffs for tiled CMP on-chip networks. ACM Int. Conf. Supercomput. 25th Anniv. Vol..

[36] Wang H., Peh L.S., Malik S. (2003). Power-driven design of router microarchitectures in on-chip networks. Proc. Annu. Int. Symp. Microarchitecture, MICRO.

[37] Mans B. (1999). On the interval routing of chordal rings. Proc. Fourth Int. Symp. Parallel Archit. Algorithms, Networks.

[38] Narayanan L., Opatrny J. (1999). Compact routing on chordal rings of degree 4. Algorithmica.

[39] Monakhova E.A. (1981). On the analytical description of the optimal two-dimensional Diophantine structures of homogeneous computing systems. Comput. Syst. Quest. Theory Constr. Comput. Syst..

[40] (2018). Cyclone V Device Overview, CV-51001.

[41] Godsil C., Royle G. (2001). Algebraic Graph Theory.

[42] Khan S., Anjum S., Gulzari U.A., Torres F.S. (2018). Comparative analysis of network-on-chip simulation tools. IET Comput. Digital Tech..

[43] Ben-Itzhak Y., Zahavi E., Cidon I., Kolodny A. (2012). HNOCS: modular open-source simulator for Heterogeneous NoCs. 2012 Int. Conf. Embed. Comput. Syst., IEEE.

[44] Kamali H.M., Hessabi S. (2016). AdapNoC: a fast and flexible FPGA-based NoC simulator. 2016 26th Int. Conf. F. Program. Log. Appl., IEEE.

